# Gastroprotective Efficacy and Safety Evaluation of Scoparone Derivatives on Experimentally Induced Gastric Lesions in Rodents

**DOI:** 10.3390/nu7031945

**Published:** 2015-03-13

**Authors:** Dong Ju Son, Gyung Rak Lee, Sungil Oh, Sung Eun Lee, Won Sik Choi

**Affiliations:** 1 School of Applied Bioscience, Kyungpook National University, Daegu 702-701, Koreas; E-Mail: sondj1@hotmail.com; 2 Department of Biotechnology, College of Natural Sciences, Soonchunhyang University, Asan, Chungnam 336-745, Korea; E-Mails: jjanglkr@nate.com (G.R.L.); jmosungil@naver.com (S.O.)

**Keywords:** scoparone, 5,6,7-trimethoxycoumarin, 6,7,8-trimethoxycoumarin, gastric ulcer, peptic ulcer, gastric mucus

## Abstract

This study investigated the gastroprotective efficacy of synthesized scoparone derivatives on experimentally induced gastritis and their toxicological safety. Six scoparone derivatives were synthesized and screened for gastroprotective activities against HCl/ethanol- and indomethacin-induced gastric ulcers in rats. Among these compounds, 5,6,7-trimethoxycoumarin and 6,7,8-trimethoxycoumarin were found to have gastroprotective activity greater than the standard drug rebamipide; 6-methoxy-7,8-methylenedioxycoumarin, 6-methoxy-7,8-(1-methoxy)-methylenedioxycoumarin, 6,7-methylenedioxycoumarin, and 6,7-(1-methoxy)-methylenedioxycoumarin were found to be equipotent or less potent that of rebamipide. Pharmacological studies suggest that the presence of a methoxy group at position C-5 or C-8 of the scoparone’s phenyl ring significantly improves gastroprotective activity, whereas the presence of a dioxolane ring at C-6, C-7, or C-8 was found to have decreased activity. In order to assess toxicological safety, two of the potent gastroprotective scoparone derivatives—5,6,7-trimethoxycoumarin and 6,7,8-trimethoxycoumarin—were examined for their acute toxicity in mice as well as their effect on cytochrome P450 (CYP) enzyme activity. These two compounds showed low acute oral toxicity in adult male and female mice, and caused minimal changes to CYP3A4 and CYP2C9 enzyme activity. These results indicate that compared to other scoparone derivatives, 5,6,7-trimethoxycoumarin and 6,7,8-trimethoxycoumarin can improve gastroprotective effects, and they have low toxicity and minimal effects on drug-metabolizing enzymes.

## 1. Introduction

The digestive system performs the essential function of breaking down food and delivering nutrients to every cell in the human body. The stomach is a major organ of the upper part of the digestive system, and it churns food to help break it down mechanically as well as chemically. It is well known that stomach disease might cause indigestion and dyspepsia, and may lead to forms of malnutrition and weight loss [1]. Gastric ulcer (GU), also known as peptic ulcer, forms in the stomach or upper part of the small intestine and is the most frequent upper gastrointestinal acid-related disease of the digestive system, significantly affecting millions of people worldwide [2,3]. GU is predominantly characterized by damage to the gastric mucosa in the stomach lining, resulting in abdominal pain, possible bleeding, and other gastrointestinal symptoms. The multifactorial etiology of GU includes bacterial infection, excessive alcohol intake, emotional stress, free radicals, the use of steroidal and non-steroidal anti-inflammatory drugs (NSAIDs), and nutritional deficiencies that disrupt the gastric mucosal barrier and make it vulnerable to normal gastric secretions [4,5].

Coumarin compounds, a large class of lactones, are medicinal agents that have attracted interest due to their potential for preventing and treating diseases having a wide range of biological activities; amazingly, these compounds have shown anti-coagulant [6,7,8], anti-neurodegenerative [9,10,11], anti-oxidant [12,13,14,15], anti-cancer [15,16,17,18,19,20], anti-microbial [21,22,23], anti-parasitic [24,25,26], and anti-inflammatory [27,28,29,30] efficacies. Recent studies have also demonstrated that coumarin compounds possess gastrointestinal protective properties [31,32,33,34]. We also previously reported that scoparone (6,7-dimethoxycoumarin), a coumarin compound from *Hericium erinaceus* cultivated with *Artemisia capillaries*, possesses an effective gastroprotective effect on gastric lesion induced by HCl/ethanol in rats [35]. These findings suggested that scoparone may be useful as a lead compound or new agent for the prevention and treatment of gastrointestinal diseases. Thus, in the present study, several scoparone derivatives were synthesized and their gastroprotective effect against chemically induced GU and toxicological safety was investigated.

## 2. Experimental Section

### 2.1. Reagents and Materials

Scoparone (**1**) was isolated and identified as previously reported [35,36]. 3,4,5-trimethoxyphenol, 7,8-dihydroxy-6-methoxycoumarin, diiodomethane, cesium carbonate, methyl (triphenylphosphoranylidence)acetate, 6,7-dihydroxycoumrin, potassium carbonate, dimethyl sulfate, titanium chloride, dichloromethyl methyl ester, ammonium chloride, absolute ethanol, hydrochloric acid, indomethacin, alcian blue dye, and formalin were purchased from Sigma-Aldrich (St. Louis, MO, USA). Rebamipide and omeprazole were purchased from Kyongbo Pharmaceutical Co. (Asan, Chungnam, Korea). All chemicals were of analytical grade.

### 2.2. Synthesis and Spectral Data of Scoparone Derivatives

#### 2.2.1. 5,6,7-Trimehoxycoumarin

Titanium chloride (135.7 mmol, 14.9 mL) was added dropwise to a stirred solution of 3,4,5-trimethoxyphenol (27.1 mmol, 5.0 g) in dichloromethane (100 mL) at 0 °C. Then the mixture was further stirred for 1 h. Dichloromethyl methyl ether (27.1 mmol, 2.4 mL) was added to this mixture and stirred at room temperature for 2 h. Saturated ammonium chloride solution was then added and reaction mixture was stirred at room temperature for 30 min. The organic phase was separated and washed with 0.1 N HCl solution (60 mL), saturated ammonium chloride solution (100 mL), and saturated hydrochloride (100 mL). The filtered solution was dried (magnesium sulfate) and evaporated under reduced pressure, and the residue was dissolved in *N*,*N*-diethylaniline (280 mL). After the addition of methyl (triphenylphosphoranylidene) acetate (22.6 mmol, 7.56 g), the reaction mixture was refluxed for 4 h. The organic phase was washed with H_2_O (400 mL) and saturated hydrochloride (200 mL), and then the filtered solution was dried and recrystallized from ethyl acetate. 5,6,7-Trimethoxycoumarin (**2**) was obtained as a pale yellow solid (1.68 g): yield 37.8%; mp 73–74 °C; IR (cm^−1^): 1032, 1105 (C–O), 1561 (C=C), 1720 (C=O); MS (*m/z*): 236 (M^+^, 100%), 221, 206, 191, 163, 135, 120, 89, 79, 69, 59, 51. ^13^C-NMR (δ ppm, DMSO) and ^1^H-NMR (δ ppm, DMSO) chemical shifts values are shown in Table 1 and Table 2, respectively.

#### 2.2.2. 6,7,8-Trimethoxycoumarin

Dimethyl sulfate (19.2 mmol, 1.82 mL) and potassium carbonate (19.2 mmol, 2.65 g) were added to a stirred solution of 7,8-dihydroxy-6-methoxycoumarin (4.8 mmol, 1.0 g) in acetone (10 mL), and then the mixture was refluxed for 3 h. Distilled water (20 mL) and ethyl acetate (20 mL) were added and stirred for 30 min, and the organic phase was washed with H_2_O (20 mL), dried (magnesium sulfate) and evaporated under reduced pressure, and residue was dissolved in isopropanol (2 mL). The filtered solution was dried and recrystallized from isopropanol. 6,7,8-trimethoxycoumarin (**3**) was obtained as a pale yellow solid (0.99 g): yield 86.9%; mp 104–105 °C; IR (cm^−1^): 1044, 1192 (C–O), 1566 (aromatic C=C), 1715 (C=O); MS (*m/z*): 236 (M^+^, 100%), 221, 191, 163, 135, 120, 89, 79, 69, 51; ^13^C-NMR (δ ppm, DMSO) and ^1^H-NMR (δ ppm, acetone) chemical shifts values are shown in Table 1 and Table 2, respectively.

#### 2.2.3. 6-Methoxy-7,8-methylenedioxycoumarin

Cesium carbonate (7.2 mmol, 2.34 g) was added to a stirred solution of 7,8-dihydroxy-6-methoxycoumarin (4.8 mmol, 1.0 g) in *N*,*N*-dimethylformamide (30 mL), and then the mixture was further stirred for 10 min. Diiodomethane (7.2 mmol, 0.58 mL) was added to this mixture and stirred at 110 °C for 1.5 h. After cooling, H_2_O (30 mL) and methylene chloride (30 mL) were added and stirred for 30 min. The organic phase was then washed with H_2_O (30 mL, 3 times), dried (magnesium sulfate) and filtered. The evaporated residue was recrystallized from methylene chloride and *n*-hexane. 6-methoxy-7,8-methylenedioxycoumarin (**4**) was obtained as a pale yellow solid (0.82 g): yield 77.5%; mp 220–221 °C; IR (cm^−1^): 1060, 1192 (C–O), 1583 (aromatic C=C), 1718 (C=O); MS (*m/z*): 220 (M^+^), 206 (100%), 191, 163, 135, 89, 79. ^13^C-NMR (δ ppm, CDCl_3_) and ^1^H-NMR (δ ppm, CDCl_3_) chemical shifts values are shown in Table 1 and Table 2, respectively.

**Table 1:** ^13^C-NMR (carbon nuclear magnetic resonance) chemical shifts of the scoparone derivatives.

C*	Compound					
	2	3	4	5	6	7
C-2	160.15	160.52	159.81	159.74	161.26	161.35
C-3	112.08	115.70	114.44	114.44	113.97	114.28
C-4	137.68	144.73	143.63	138.24	140.78	143.30
C-5	148.73	105.48	105.05	104.76	112.68	112.43
C-6	139.06	151.15	141.11	143.72	143.51	144.54
C-7	156.93	143.72	133.97	133.31	144.91	152.02
C-8	95.94	146.76	135.05	140.48	105.03	103.59
C-9	150.90	141.83	139.97	133.48	151.24	150.55
C-10	106.43	115.50	114.33	114.23	113.39	113.34
C-11	56.42	56.60	56.82	56.83	102.36	121.53
C-12	60.71	61.92	103.60	121.55	-	50.81
C-13	61.81	61.54	-	50.64	-	-

**Figure 1:**
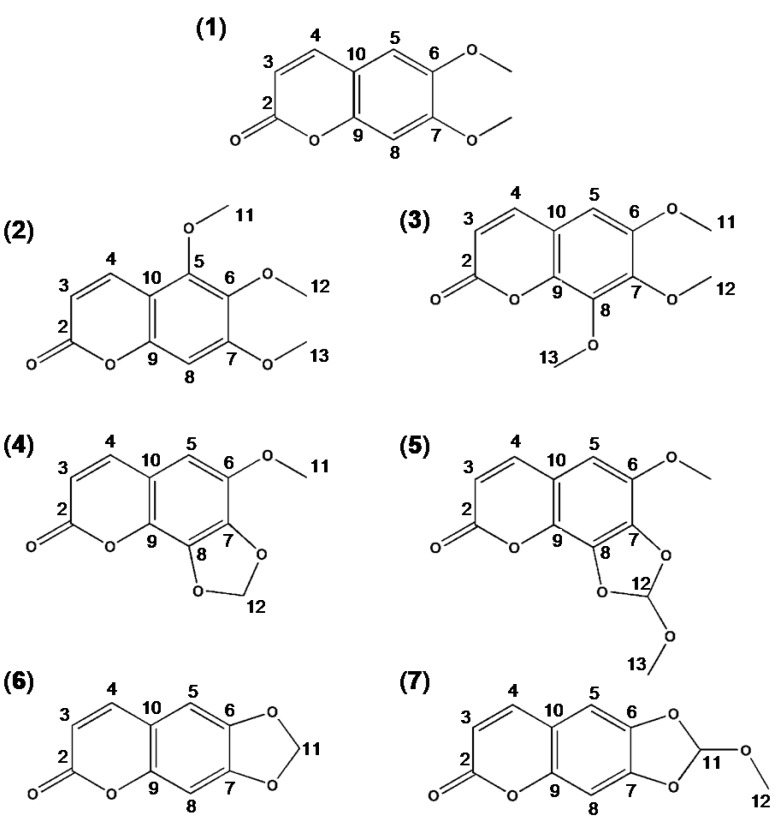
Structure of scoparone and its synthetic derivatives. Scoparone (6,7-dimethoxycoumarin) (**1**), 5,6,7-trimethoxycomarin (**2**), 6,7,8-trimethoxycoumarin (**3**), 6-methoxy-7,8-methylenedioxycoumarin (**4**), 6-methoxy-7,8-(1-methoxy)-methylenedioxycoumarin (**5**), 6,7-methylenedioxycoumarin (**6**), 6,7-(1-methoxy)-methylenedioxycoumarin (**7**).

* Carbon numbering given in Figure 1.

**Table 2:** ^1^H-NMR (proton nuclear magnetic resonance) chemical shifts of the scoparone derivatives.

C*	Compound					
	2	3	4	5	6	7
C-2	-	-	-	-	-	-
C-3	6.23, 6.25(d, 1H)	6.30, 6.33(d, 1H)	6.26, 6.29(d, 1H)	6.28, 6.31(d, 1H)	6.25, 6.29(d, 1H)	6.15, 6.30(d, 1H)
C-4	7.95, 7.97(d, 1H)	7.87, 7.89(d, 1H)	7.57, 7.59(d, 1H)	7.59, 7.61(d, 1H)	7.57, 7.60(d, 1H)	7.79, 7.93(d, 1H)
C-5	-	7.03(s, 1H)	6.59(s, 1H)	6.61(s, 1H)	6.83(s, 1H)	6.59(s, 1H)
C-6	-	-	-	-	-	-
C-7	-	-	-	-	-	-
C-8	6.86(s, 1H)	-	-	-	6.96(s, 1H)	6.80(s, 1H)
C-9	-	-	-	-	-	-
C-10	-	-	-	-	-	-
C-11	3.75(s, 3H)	3.89(s, 3H)	-	3.95(s, 3H)	6.09(s, 2H)	7.06(s, 1H)
C-12	3.88(s, 3H)	3.93(s, 3H)	5.17(s, 2H)	7.04(s, 1H)	-	3.45(s, 3H)
C-13	3.93(s, 3H)	3.97(s, 3H)	-	3.49(s, 3H)	-	-

* According to carbon numbering given in Figure 1.

#### 2.2.4. 6-Methoxy-7,8-(1-methoxy) Methylenedioxycomarin

Cesium carbonate (7.2 mmol, 2.34 g) was added to a stirred solution of 7,8-dihydroxy-6-methoxycoumarin (4.8 mmol, 1.0 g) in *N*,*N*-dimethylformamide (30 mL), and then the mixture was further stirred for 10 min. Dichloromethyl methyl ether (7.2 mmol, 0.63 mL) was added to this mixture and stirred at room temperature for 5 h. Saturated sodium bicarbonate solution (30 mL) and methylene chloride (30 mL) were added and stirred for 30 min. The organic phase was washed with H_2_O (30 mL, 3 times), dried (magnesium sulfate), and filtered. The evaporated residue was recrystallized from methylene chloride and *n*-hexane. 6-Methoxy-7,8-(1-methoxy)methylenedioxycoumarin (**5**) was obtained as a pale yellow solid (0.84 g): yield 70.0%; mp 202–203 °C; IR (cm^−1^): 1047, 1204 (C–O), 1587 (aromatic C=C), 1716 (C=O); MS (*m/z*): 250 (M^+^), 235, 205 (100%), 191, 163, 135, 116, 79. ^13^C-NMR (δ ppm, CDCl_3_) and ^1^H-NMR (δ ppm, CDCl_3_) chemical shifts values are shown in Table 1 and Table 2, respectively.

#### 2.2.5. 6,7-Methylenedioxycomarin

Cesium carbonate (8.4 mmol, 2.74 g) was added to a stirred solution of 6,7-dihydroxycoumarin (5.6 mmol, 1.0 g) in *N*,*N*-dimethylformamide (30 mL), and then the mixture was further stirred for 10 min. Diiodomethane (8.4 mmol, 0.67 mL) was added to this mixture and stirred at 60 °C for 1.5 h. Saturated sodium bicarbonate solution (30 mL) and methylene chloride (30 mL) were added and stirred for 30 min. The organic phase was washed with H_2_O (30 mL, 3 times), dried (magnesium sulfate), and filtered. The evaporated residue was recrystallized from methylene chloride and *n*-hexane. 6,7-Methylenedioxycomarin (**6**) was obtained as a pale yellow solid (0.4 g): yield 38.0%; mp 230–231 °C; IR (cm^−1^): 1035, 1161 (C–O), 1581 (aromatic C=C), 1715 (C=O); MS (*m/z*): 190 (M^+^), 178 (100%), 163, 135, 120, 89, 79, 69. ^13^C-NMR (δ ppm, CDCl_3_) and ^1^H-NMR (δ ppm, CDCl_3_) chemical shifts values are shown in Table 1 and Table 2, respectively.

#### 2.2.6. 6,7-(1-Methoxy)methylenedioxycomarin

Cesium carbonate (8.4 mmol, 2.74 g) was added to a stirred solution of 6,7-dihydroxycoumarin (5.6 mmol, 1.0 g) in *N*,*N*-dimethylformamide (30 mL), and then the mixture was further stirred for 10 min. Dichloromethyl methyl ether (8.4 mmol, 0.74 mL) was added to this mixture and stirred at room temperature for 5 h. Saturated sodium bicarbonate solution (30 mL) and methylene chloride (30 mL) were added and stirred for 30 min. The organic phase was washed with H_2_O (30 mL, 3 times), dried (magnesium sulfate), and filtered. The evaporated residue was recrystallized from methylene chloride and *n*-hexane. 6,7-(1-Methoxy)methylenedioxycoumarin (**7**) was obtained as a pale yellow solid (0.85 g): yield 69.0%; mp 205–206 °C; IR (cm^−1^): 1054, 1192 (C–O), 1556 (aromatic C=C), 1712 (C=O); MS (*m/z*): 220(M^+^), 215, 189, 178 (100%), 163, 135, 120, 79. ^13^C-NMR (δ ppm, acetone) and ^1^H-NMR (δ ppm, acetone) chemical shifts values are shown in Table 1 and Table 2, respectively.

### 2.3. Animals and Ethics Statement

Sprague-Dawley (S.D.) rats (9 weeks old) and ICR mice (6 weeks old) were obtained from Taconic Korea (Daehan Biolink Co. Ltd., Umsung, Chungbuk, Korea). Animals were maintained under conventional housing conditions at 23 ± 2 °C with a controlled 12 h light/dark cycle, and drinking water and rodent chow diet were provided *ad libitum* throughout the experiment, except for the 12 h fasting period prior to treatment where the drinking water was still in free access but no diet supply was provided. All animal experiments were conducted in accordance with the principles and procedures outlined in the National Institute of Health Guide of the Care and Use of Laboratory Animals. The protocols for animal experiments were approved by the animal ethics committee at Soonchunhyang University (Ethics No. SCH13-01-02 and SCH13-01-03).

### 2.4. Gastroprotective Efficacy Assessments

#### 2.4.1. HCl/Ethanol-Induced Gastritis in Rats

The male S.D. rats were fasted for 24 h prior to oral dosing with the normal saline (vehicle), rebamipide (a positive control), or test compounds (Figure 1) at a range of doses, which are described in the results. One hour after the treatments, all animals orally received 0.5 mL of a mixture of 0.15 M HCl and 60% ethanol solution. Animals were sacrificed by cervical dislocation 3 h after the administration of HCl/ethanol solution, the stomach was removed and fixed in 4% formalin solution for 1 h, opened along the greater curvature, and photographed using a camera attached to a dissection microscope. Total area (mm^2^) of mucosal erosive lesion was measured using Photoshop CS4 Extended (Adobe, San Jose, CA, USA) software. The percentage of ulcer inhibition by test compounds was calculated by the following formula:

% protection of ulceration = ((%U_area_ vehicle treated ulcer control–%U_area_ compound treated)/%U_area_ vehicle treated ulcer control) × 100 (1)

where *%U_area_* is the percent ulcer area of the total gastric mucosa area. ED_50_ values (50% protection against GU) were calculated from the dose-response curves of logarithmic plots of each test compound.

#### 2.4.2. Indomethacin-Induced Gastritis in Rats

The male S.D. rats were fasted for 24 h prior to oral dosing with normal saline, rebamipide, or test compounds at a dose of 40 mg/kg body weight. One hour after treatment, all animals orally received 0.5 mL indomethacin solution (80 mg/kg body weight, suspended in 5% sodium bicarbonate). Animals were sacrificed by cervical dislocation 6 h after the administration of indomethacin solution, the stomach was removed and fixed in 2% formalin solution for 30 min, opened along the greater curvature, and photographed using a camera attached to a dissection microscope. Total area (mm^2^) of mucosal erosive lesion was measured and the % protection was calculated as described above.

#### 2.4.3. Measurement of Adherent Gastric Mucus

The male S.D. rats were fasted for 24 h prior to oral dosing with normal saline, rebamipide, or test compounds at a dose of 20 mg/kg body weight. One hour after the treatments, all animals orally received 0.5 mL of a mixture of 0.15 M HCl and 60% ethanol solution. Animals were sacrificed by cervical dislocation 3 h after the administration of HCl/ethanol solution, the stomach was removed, and parts of the stomach mucosa were rinsed with ice-cold 0.25 M sucrose. Measurement of adherent gastric mucosal mucus was assayed using alcian blue, a dye which stains acid mucosubstances, as previously described [37]. In brief, a 100 mm^2^ portion of the glandular region of the stomach was excised with a scalpel, and soaked in 0.1% alcian blue dissolved in 0.16 M of sucrose buffered with 0.05 M sodium acetate (pH 5.8) for 2 h. The unbound dye was removed using two successive washes with 0.25 M sucrose. The dye complex with mucus was extracted using 30% docusate sodium salt for 2 h. After centrifugation at 2600× *g* for 10 min, the optical density of the alcian blue solution was measured at 620 nm and calculated using the calibration curve. The adherent gastric mucosal mucus was expressed as the amount of the alcian blue (μg/g tissue).

### 2.5. Toxicological Safety Assessments

#### 2.5.1. Acute Toxicity Studies

Acute toxicity studies were performed both in male and female ICR mice as described previously [38]. Vehicle control and test compound treated groups consisted of 10 animals each. Mice were acclimatized for 7 days before experimentation. Before dosing with test compounds, animals were fasted for 24 h with access to adequate drinking water in cases with wire mesh bottoms, to prevent coprophagy. The treated group received test compounds, and the control group received saline by gavage at a dose of 1000 mg/kg body weight. Animals were observed carefully for any indications of toxicity within the first six hours after the treatment. The mortality, body measurements, and behavior were assessed and recorded daily for 14 days after treatment. The macroscopic analyses and weight of vital organs, such as the liver, kidney, spleen, lung, and heart were compared between the animals treated with test compounds and vehicle.

#### 2.5.2. Cytochrome P450 Enzyme Activity Assay *in vitro*

The inhibition assays for human cytochrome P450 (CYP) enzyme activities were performed in multiwall plates using CYP2C9 and CYP3A4 screening kits (Invitrogen Co., Carlsbad, CA, USA) according to the manufacturer’s instruction. The test compounds Ketoconazole (a positive inhibition control for CYP3A4) or Sulfaphenazole (a positive inhibition control for CYP2C9) were mixed with each master pre-mix comprising CYP2C9 or CYP3A4 BACULOSOMES^®^ reagent and the regeneration system, which contained glucose-6-phosphate and glucose-6-phosphate dehydrogenase. The mixture was incubated at room temperature for 20 min. Following incubation, a CYP enzyme-specific substrate (Vivid BOMCC) and NADP^+^ were added and the mixture was incubated at room temperature for 30 min. The reaction was stopped by the addition of a Stop Reagent (CYP450 isozyme specific inhibitors). CYP activity was evaluated by measuring the fluorescence of the fluorescent metabolite generated from each CYP enzyme-specific substrate. The fluorescence was measured using a fluorescent plate reader at 409 nm (excitation) and 460 nm (emission). All experiments were performed in duplicate, and the results are expressed as percent inhibition.

### 2.6. Statistical Analysis

Statistical analyses were carried out with Graph-Pad Prism (GraphPad Software). Pairwise comparisons were performed using one-way Student’s *t*-tests. Data are presented as means ± standard error of the mean (S.E.M) in the indicated number of experiments. Differences between groups were considered significant at *p*-values below 0.05.

## 3. Results

### 3.1. Effect of Scoparone Derivatives on Gastric Ulcer

**Figure 2:**
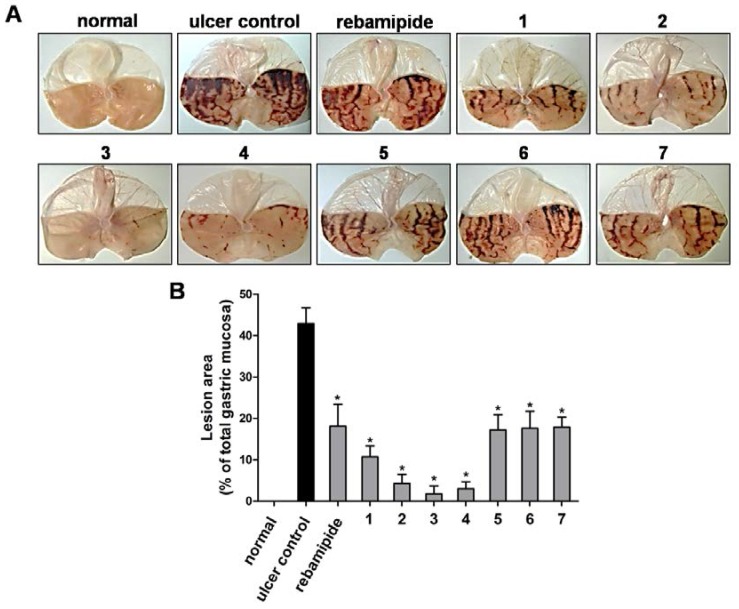
The effect of scoparone and its synthetic derivatives on HCl/ethanol-induced gastric ulcers. (**A**) Rats treated with normal saline, as the vehicle, show no injuries to the gastric mucosa (normal). Rats pretreated with normal saline followed by oral administration of HCl/ethanol solution. Severe injuries and hemorrhage necrosis are seen in the gastric mucosa (ulcer control). Rats pretreated with a 40 mg/kg dose of rebamipide, scoparone (**1**), 5,6,7-trimethoxycomarin (**2**), 6,7,8-trimethoxycoumarin (**3**), 6-methoxy-7,8-methylenedioxycoumarin (**4**), 6-methoxy-7,8-(1-methoxy)-methylenedioxycoumarin (**5**), 6,7-methylenedioxycoumarin (**6**), or 6,7-(1-methoxy)-methylenedioxycoumarin (**7**) followed by oral administration of HCl/ethanol solution. Stomachs were dissected, and imaged by bright field microscopy. Representative images of the each experimental group are shown; (**B**) Gastric ulcer lesion area was quantified. Data shown as mean ± SEM of (*n* = 8). *p < 0.05 *vs.* ulcer control as determined by Student’s *t*-test.

Initially, we carried out a functional activity assay to evaluate the gastroprotective activity of synthesized scoparone derivatives on HCl/ethanol-induced GU animal model treated with a single oral dose of compound (40 mg/kg body weight in female S.D. rats). As contrasted with normal control group, oral administration of HCl/ethanol solution to the ulcer control group clearly produced characteristic hemorrhagic lesions with large liner patches of mucosal necrosis and edema that were significantly protected by scoparone (**1**) treatment (Figure 2A), consistent with our previous observation [35]. Under our experimental condition, we confirmed that rebamipide, a currently used medicine for gastritis treatment, significantly decreases the appearance of gastric lesion with a protection rate of 57.8%. We found that treatment with 5,6,7-trimethoxycoumarin (**2**), 6,7,8-trimethoxycoumarin (**3**), and 6-methoxy-7,8-methylene-dioxycoumarin (**4**) exhibited very strong gastroprotective activities compared to rebamipide, with protection rates of 90.0%, 95.8%, and 93.1%, respectively (Figure 2). In contrast, the gastroprotective activities of 6-methoxy-7,8-(1-methoxy)-methylenedioxycoumarin (**5**), 6,7-methylenedioxycoumarin (**6**), and 6,7-(1-methoxy)methylenedioxycoumarin (**7**) were similar to rebamipide treatment (Figure 2). We further found that treatment with a dose of 40 mg/kg of scoparone (**1**), 5,6,7-trimethoxycoumarin (**2**), and 6,7,8-trimethoxycoumarin (**3**) effectively inhibited indomethacin-induced gastric damages, an NSAID drug-induced GU. However, treatment of animals with 6-methoxy-7,8-methylene-dioxycoumarin (**4**), which exhibited strong inhibitory activity against HCl/ethanol-induced GU, showed equipotent gastroprotective activity that of rebamipide on indomethacin-induced GU (Supplementary Figure S1).

**Figure 3:**
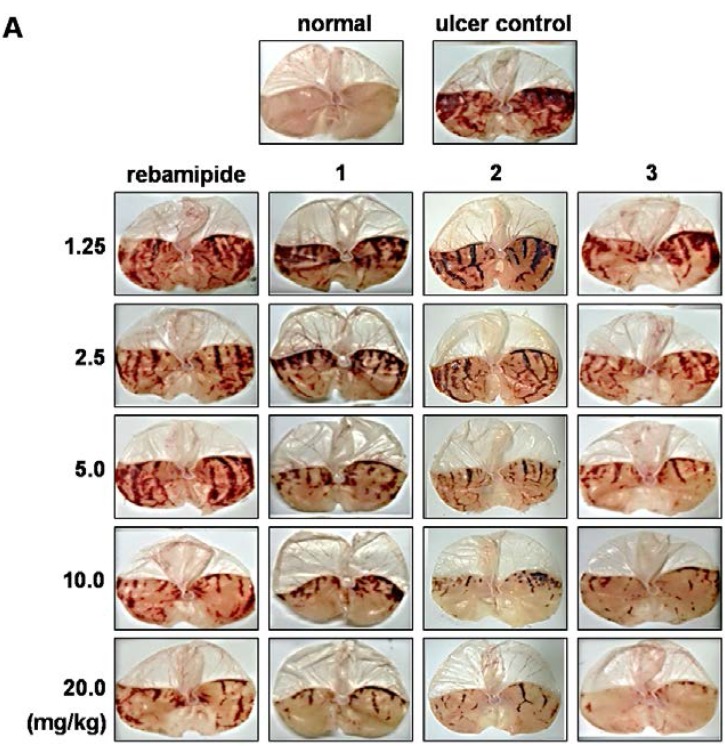

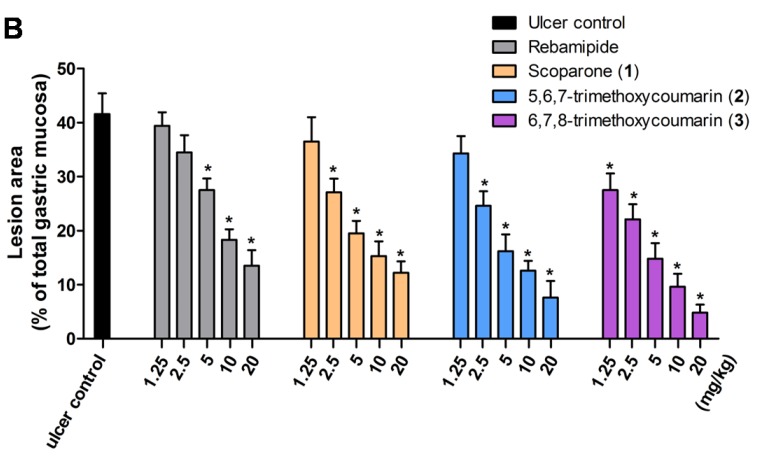
The dose-dependent effect of scoparone and its synthetic derivatives on HCl/ethanol-induced gastric ulcers. (**A**) Normal saline, as the vehicle, or normal saline and HCl/ethanol solution treated rats were used as normal control or the ulcer control, respectively. Rats were pretreated with a range of doses of rebamipide, scoparone (**1**), 5,6,7-trimethoxycomarin (**2**), or 6,7,8-trimethoxycoumarin (**3**), as presented in the figure. Rats were subsequently treated with HCl/ethanol solution. Stomachs were dissected, and imaged by bright field microscopy. A representative image of each experimental group is shown; (**B**) Gastric ulcer lesion area was quantified. Data shown as mean ± SEM of (*n* = 8). *p < 0.05 *vs.* ulcer control as determined by Student’s *t*-test.

In addition, two potent scoparone derivatives, 5,6,7-trimethoxycoumarin (**2**) and ,7,8-trimethoxycoumrin (**3**), showed a dose-dependent protective activity against HCl/ethanol-induced GU with ED_50_ values of 3.94 and 2.93 mg/kg, respectively, which are stronger than that of rebamipide (ED_50_, 9.53 mg/kg) and scoparone (**1**) (ED_50_, 4.21 mg/kg) (Figure 3). These findings demonstrated a potent and improved gastroprotective activity by synthesized scoparone derivatives, especially 5,6,7-trimethoxycoumarin (**2**) and 6,7,8-trimethoxycoumarin (**3**), compared to standard treatment in two different rodent model of GU.

### 3.2. Effect of Scoparone Derivatives on Adherent Gastric Mucus

To investigate the possible mechanism of gastroprotective activity, we tested the effect of 5,6,7-trimethoxycoumarin (**2**) and 6,7,8-trimethoxycoumarin (**3**), two scoparone derivatives that showed significantly improved gastroprotective activity compared to scoparone on HCl/ethanol-induced gastritis, on gastric mucus secretion in gastric lesion. The effects of 5,6,7-trimethoxycoumarin (**2**) and 6,7,8-trimethoxycoumarin (**3**) on the adherent mucus content of gastric mucosa are presented in Table 3. The alcian blue binding capacity of gastric wall mucus was significantly reduced in rats exposed to HCl/ethanol solution (ulcer control), which was recovered by treatment with rebamipide (1584 ± 80 μg/g of tissue), compared to normal control rats (1618 ± 64 μg alcian blue/g of tissue). We found that rats treated with 5,6,7-trimethoxycoumarin (**2**) (1424 ± 83 μg/g of tissue) and 6,7,8-trimethoxycoumarin (**3**) (1876 ± 51 μg/g of tissue) at 20 mg/kg body weight significantly increased the adherent gastric mucus content compared to the ulcer control (456 ± 74 μg/g of tissue). These findings suggest that the potent gastroprotective activities of 5,6,7-trimethoxycoumarin (**2**) and 6,7,8-trimethoxycoumarin (**3**) may be mediated by induced gastric mucus secretion.

**Table 3:** Effect of scoparone derivatives on adherent gastric mucus content in HCl/ethanol-treated rats.

Treatment	Dose (mg/kg body weight)	Adherent Mucus (μg alcian blue/g tissue)
Normal control	-	1618 ± 64^*^
Ulcer control	-	456 ± 74^*^
Rebamipide	20	1584 ± 80 *
5,6,7-trimethoxycoumarin (**2**)	20	1424 ± 83 *
6,7,8-trimethoxycoumarin (**3**)	20	1876 ± 51 *

Rats were pretreated with normal saline (normal control), rebamipide (positive control), 5,6,7-trimethoxycoumarin (**2**), or 6,7,8-trimethoxycoumarin (**3**) at a dose of 20 mg/kg or saline (ulcer control) one hour before they were subjected to HCl/ethanol solution treatment. The adherent gastric mucus content was measured as described in the methods. Data are shown as mean ± SEM of (*n* = 6). * *p* < 0.05 *vs.* ulcer controls as determined by Student’s *t*-test.

### 3.3. Acute Toxicity Assay Results

**Figure 4:**
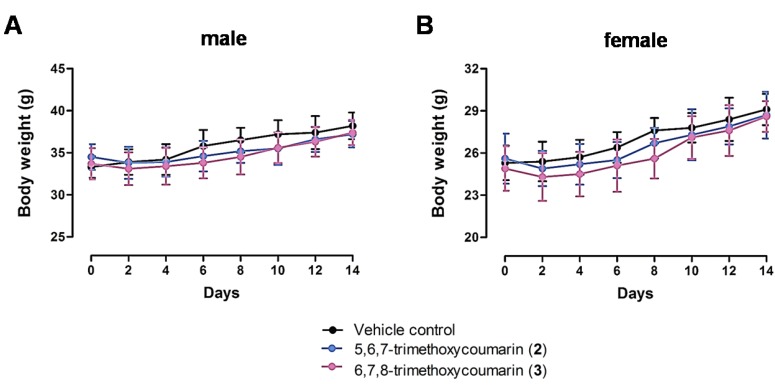
Mice body weight changes following oral administration with scoparone derivatives. Both male (**A**) and female (**B**) 7 week old ICR mice were orally administered saline (black circles) or 1000 mg/kg 5,6,7-trimethoxycomarin (**2**) (blue circles) or 6,7,8-trimethoxycoumarin (**3**) (purple triangles). Data are shown as mean ± SEM of (*n* = 10). There are no significant differences between groups.

Acute toxicities in mice treated with 5,6,7-trimethoxycoumarin (**2**) and 6,7,8-trimethoxycoumarin (**3**), two potent scoparone derivatives, were investigated. A single oral administration of test compounds at 1000 mg/kg body weight revealed no toxicity in treated ICR mice. After 14 days of administration, no animal died and no significant alteration in body weight (Figure 4) or relative weight of liver, kidney, spleen, heart, or lungs were observed in relation to the vehicle control group (Supplementary Table S1). The LD_50_ values, expressed as mg of compound per kg of mouse body weight, were calculated according to the statistical Probit method. The LD_50_ values obtained were 3812 to 3857 and 2111 to 3500 for 5,6,7-trimethoxycoumarin (**2**) and 6,7,8-trimethoxycoumarin (**3**), respectively, in adult male and female ICR mice (Table 4). These results indicate that 5,6,7-trimethoxycoumarin (**2**) and 6,7,8-trimethoxycoumarin (**4**) have low acute oral toxicity in mice.

**Table 4:** The effect of scoparone derivatives on acute oral toxicity in mice.

Treatment	LD_50_ (mg/kg body weight)	
	Male	Female
5,6,7-trimethoxycoumarin (**2**)	3812	3857
6,7,8-trimethoxycoumarin (**3**)	2111	3500

### 3.4. Effect of Scoparone Derivatives on Human Recombinant CYP Enzyme Activity

As part of a safety study, the effect of 5,6,7-trimethoxycoumarin (**2**) and 6,7,8-trimethoxycoumarin (**3**) on CYP enzyme activity was investigated. We found that CYP3A4 activity was slightly decreased by 5,6,7-trimethoxycoumarin (**2**) (19.1%) and 6,7,8-trimethoxycoumarin (**2**) (15.8%) treatment at 10 μM, and CYP2C9 activity was not affected by test compounds at the same concentration (Table 5). We further confirmed that, under our experimental condition, the CYP3A4 inhibitor ketoconazole and CYP2C9 inhibitor Sulfaphenazole specifically inhibited the enzyme activity of CYP3A4 and CYP2C9, respectively. These results suggest that 5,6,7-trimethoxycoumarin (**2**) and 6,7,8-trimethoxycoumarin (**3**) may have a low risk of drug-drug interaction.

**Table 5:** Effect of scoparone derivatives on the activity of human recombinant cytochrome P450 (CYP) enzymes.

Treatment	Concentration (μM)	Inhibition (%)	
		CYP3A4	CYP2C9
Ketoconazole	20	97.5	0
Sulfaphenazole	20	0	93.4
Scoparone (**1**)	10	32.7	0
5,6,7-trimethoxycoumarin (**2**)	10	19.1	0
6,7,8-trimethoxycoumarin (**3**)	10	15.8	0

## 4. Discussion

In present study, scoparone derivatives were synthesized and their gastroprotective efficacy, acute toxicity, and drug-drug interaction potential were evaluated in rodents. We demonstrated that 5,6,7-trimethoxycourmarine (**2**) and 6,7,8-trimethoxycoumarin (**3**), which have the electron donating substituent (-OCH_3_) at position C-5 or C-8 of the scoparone’s phenyl ring, strongly improve gastroprotective activity in both HCl/ethanol- and indomethacin-induced GU rat models. Additionally, we demonstrated that these chemically modified scoparone derivatives protect the gastric mucosal surface by significantly inducing gastric mucus secretion in rats. We also found that 5,6,7-trimethoxycourmarine (**2**) and 6,7,8-trimethoxycoumarin (**3**) have low acute oral toxicity in mice, and cause minimal changes to human CYP enzyme activity.

Scoparone (**1**) is a coumarin compound that has been reported to exhibit diverse biological actions, including hepato-protective [39,40,41,42], anti-inflammation [12,43,44], anti-cancer [45,46], anti-neurodegenerative [47,48], and anti-oxidant [12,49] effects. We previously reported that scoparone (**1**) provides a gastroprotective effect in HCl/ethanol-induced GU rat model [35]. On this basis, we directed our attention to the chemical modification of scoparone (**1**) with the aim of improving gastroprotective activity (Figure 1). In the present study, we found that two scoparone derivatives, 5,6,7-trimethoxycourmarine (**2**) and 6,7,8-trimethoxycoumarin (**3**), significantly improve gastroprotective activity against both HCl/ethanol- and indomethacin-induced GU compared to scoparone (**1**), resulting in over 90% GU reversal. This suggests that the substituents at the scoparone moiety, particularly those at positions C-5 and C-8, influence the gastroprotective activity in a significant manner. Electron-donating groups, such as -OCH_3_, -OH, and -NH_2_, are known to increase biological activity and have generally been associated with an increase in the lipophilicity of compounds [50]. This might explain the improved gastroprotective activity of 5,6,7-trimethoxycoumarin (**2**) and 6,7,8-trimethoxycoumarin (**3**), which have an -OCH_3_ group at position C-5 or C-8 of the scoparone’s phenyl ring, respectively, compared to scoparone (**1**). Conversely, other scoparone derivatives containing a dioxolane ring at position C-6, C-7, or C-8 showed significantly reduced gastroprotective activity even though 6-methoxy-7,8-methylenedioxycoumarin (**4**), which has a dioxolane ring as well as an -OCH_3_ group at position C-6, effectively inhibited HCl/ethanol-induced GU but not indomethacin-induced GU. This result suggested that the presence of bulkier substituents containing a dioxolane ring at position C-6, C-7, or C-8 may be associated with significantly reduced gastroprotective efficacy. This indicates the critical role of electronic as well as steric effects on gastroprotective activity. Taken together, our findings demonstrated that the introduction of a methoxy group at the position C-5 or C-6 of the scoparone’s phenyl ring improves the gastroprotective efficacy of scoparone (**1**).

Ulcerogenic risk factors, such as excessive alcohol consumption and use of NSAID drugs, cause dispersal of the protective mucus gel and the phospholipid bilayer, resulting in acid back diffusion and mucosal injury secretions. The first line of defense against acid is the gastric mucus, which together with bicarbonate, covers the entire gastric mucosa and protects against ulcerogenic factors [4,5,51]. Scoparone (**1**) is known to stimulate airway and intestinal mucosal secretion [52]. However, scoparone (**1**) and its derivatives have never been evaluated for their effect on gastric mucus secretion. In the present study, we demonstrated that S.D. rats treated with HCL/ethanol solution show a significant reduction in gastric wall mucus level, and this was effectively reversed by oral administration of 5,6,7-trimethoxycoumarin (**2**) and 6,7,8-trimethoxycoumarin (**3**). This suggests that 5,6,7-trimethoxycoumarin (**2**) and 6,7,8-trimethoxycoumarin (**3**) might protect against GU by increasing gastric mucus secretion from gastric mucosal cells.

The magnitude of the therapeutic *versus* toxicological effects of a drug is a vital parameter in assessing a drug’s applicability in the clinic. As part of this pharmacological study, 5,6,7-trimethoxycoumarin (**2**) and 6,7,8-trimethoxycoumarin (**3**) were investigated for the acute toxicity. Our acute toxicity study showed that 5,6,7-trimethoxycoumarin (**2**) and 6,7,8-trimethoxycoumarin (**3**) were safe when given as a single dose by oral gavage to mice at 1000 mg/kg body weight. Throughout the experimental period, there were no clinical signs or gross findings indicating treatment-related adverse effects in any of the 5,6,7-trimethoxycoumarin (**2**) and 6,7,8-trimethoxycoumarin (**3**) treated mice. Although clinical indications like body weight loss were observed in some mice, these symptoms generally occurred spontaneously in the toxicity test due to systemic administration [53]. Additionally, these symptoms were not statistically significant. Therefore, this symptom was not considered to be a 5,6,7-trimethoxycoumarin (**2**) or 6,7,8-trimethoxycoumarin (**3**) treatment-related abnormality. In addition, no gross findings and organ weight changes were observed in same animals. Based on these results, 5,6,7-trimethoxycoumarin (**2**) and 6,7,8-trimethoxycoumarin (**3**) are extrapolated to offer a wide margin of safety by oral administration. However, since toxicity in animals and humans is genetically diverse and may respond differently in particular individuals, especially with respect to people with gastrointestinal disorders, additional toxicological assessments need to be performed to evaluate the safety of 5,6,7-trimethoxycoumarin (**2**) and 6,7,8-trimethoxycoumarin (**3**).

Interaction of drug with another drug (drug-drug interaction), food, or dietary supplement (drug-food/nutrition interaction) represents a major safety concern in drug treatment [54,55]. CYPs generally convert a large number of exogenous compounds (toxic, carcinogenic, and most pharmaceutical agents) to less toxic compounds. CYP enzymes are mainly expressed in the human liver and gastrointestinal tract and metabolize many pharmacologic agents and chemicals. It is well known that CYP3A4 is one of the dominant CYP enzymes in both the liver and extra-hepatic tissues, and it plays an important role in the oxidation of xenobiotics and contributes to the metabolism of about 60% of currently used therapeutic drugs [56,57]. It is also well established that CYP2C9 is one of the major CYP enzymes involved in the metabolism of a wide range of therapeutic agents, fatty acids, prostanoids, and steroid hormones [58,59]. Therefore, induction or inhibition of CYP3A4 or CYP2C9 enzyme activity is important for the identification of potential drug-drug interactions or toxicity of drugs in humans [60,61,62]. We investigated the effect of 5,6,7-trimethoxycoumarin (**2**) and 6,7,8-trimethoxycoumarin (**3**) on the enzyme activity of human CYP3A4 and CYP2C9 *in vitro*. We found that 5,6,7-trimethoxycoumarin (**2**) and 6,7,8-trimethoxycoumarin (**3**) only produced 19.1% and 15.8% inhibition, respectively, on CYP3A4 metabolism while scoparone (**1**) produced a 32.7% inhibition at the same concentration. We also found that 5,6,7-trimethoxycoumarin (**2**) and 6,7,8-trimethoxycoumarin (**3**) have no affect on CYP2C9 enzyme activity. These results indicate that the drug-drug interaction potential of 5,6,7-trimethoxycoumarin (**2**) and 6,7,8-trimethoxycoumarin (**3**) with CYP3A4 and CYP2C9 substrates is very low.

It has previously been reported that 5,6,7-trimethoxycoumarin (**2**) [63,64,65,66,67,68,69,70] and 6,7,8-trimethoxycoumarin (**3**) [71,72,73,74,75] can be isolated from several medicinal plants, but its biological activities remain poorly understood to date. To the best of our knowledge, this is the first comprehensive study on the synthesis of scoparone derivatives and their gastroprotective activity and toxicological safety.

## 5. Conclusions

In conclusion, among the tested scoparone synthetic derivatives, 5,6,7-trimethoxycoumarin and 6,7,8-trimethoxycoumarin have great potential for use in the protection against and treatment of GU with low acute oral toxicity and drug-drug interaction potential. Therefore, 5,6,7-trimethoxycoumarin and 6,7,8-trimethoxycoumarin might have potential for further development as a safe and effective therapeutic agent in protecting and treating gastrointestinal disorders. Further studies are warranted to determine the exact gastroprotective mechanism of 5,6,7-trimethoxycoumarin and 6,7,8-trimethoxycoumarin, and to investigate how they can be used in gastrointestinal disease therapies.

## Supplementary Files

Supplementary File 1

## References

[1] Rogers K. (2011). The Digestive System.

[2] El-Serag H.B. (2007). Time trends of gastroesophageal reflux disease: A systematic review. Clin. Gastroenterol. Hepatol..

[3] Schubert M.L., Peura D.A. (2008). Control of gastric acid secretion in health and disease. Gastroenterology.

[4] Saxena B., Krishnamurthy S., Singh S. (2011). Gastroprotective potential of risperidone, an atypical antipsychotic, against stress and pyloric ligation induced gastric lesions. Chem. Biol. Interact..

[5] Sostres C., Gargallo C.J., Lanas A. (2014). Interaction between infection, nonsteroidal anti-inflammatory drugs and/or low-dose aspirin use: Old question new insights. World J. Gastroenterol..

[6] Verhoef T.I., Redekop W.K., Daly A.K., van Schie R.M., de Boer A., Maitland-van der Zee A.H. (2014). Pharmacogenetic-guided dosing of coumarin anticoagulants: Algorithms for warfarin, acenocoumarol and phenprocoumon. Br. J. Clin. Pharmacol..

[7] Jain M., Surin W.R., Misra A., Prakash P., Singh V., Khanna V., Kumar S., Siddiqui H.H., Raj K., Barthwal M.K. (2013). Antithrombotic activity of a newly synthesized coumarin derivative 3-(5-hydroxy-2,2-dimethyl-chroman-6-yl)-*n*-{2-[3-(5-hydroxy-2,2-dimethyl-chroman-6 -yl)-propionylamino]-ethyl}-propionamide. Chem. Biol. Drug Des..

[8] Peng X.M., Damu G.L., Zhou C. (2013). Current developments of coumarin compounds in medicinal chemistry. Curr. Pharm. Des..

[9] Asadipour A., Alipour M., Jafari M., Khoobi M., Emami S., Nadri H., Sakhteman A., Moradi A., Sheibani V., Homayouni Moghadam F. (2013). Novel coumarin-3-carboxamides bearing *N*-benzylpiperidine moiety as potent acetylcholinesterase inhibitors. Eur. J. Med. Chem..

[10] Huang L., Su T., Li X. (2013). Natural products as sources of new lead compounds for the treatment of Alzheimer’s disease. Curr. Top. Med. Chem..

[11] Patil P.O., Bari S.B., Firke S.D., Deshmukh P.K., Donda S.T., Patil D.A. (2013). A comprehensive review on synthesis and designing aspects of coumarin derivatives as monoamine oxidase inhibitors for depression and Alzheimer’s disease. Bioorg. Med. Chem..

[12] Witaicenis A., Seito L.N., da Silveira Chagas A., de Almeida L.D., Luchini A.C., Rodrigues-Orsi P., Cestari S.H., di Stasi L.C. (2014). Antioxidant and intestinal anti-inflammatory effects of plant-derived coumarin derivatives. Phytomedicine.

[13] Guinez R.F., Matos M.J., Vazquez-Rodriguez S., Santana L., Uriarte E., Olea-Azar C., Maya J.D. (2013). Synthesis and evaluation of antioxidant and trypanocidal properties of a selected series of coumarin derivatives. Future Med. Chem..

[14] Bubols G.B., Vianna Dda R., Medina-Remon A., von Poser G., Lamuela-Raventos R.M., Eifler-Lima V.L., Garcia S.C. (2013). The antioxidant activity of coumarins and flavonoids. Mini Rev. Med. Chem..

[15] Kang Y.F., Liu C.M., Kao C.L., Chen C.Y. (2014). Antioxidant and anticancer constituents from the leaves of liriodendron tulipifera. Molecules.

[16] Lin M.H., Cheng C.H., Chen K.C., Lee W.T., Wang Y.F., Xiao C.Q., Lin C.W. (2014). Induction of ros-independent jnk-activation-mediated apoptosis by a novel coumarin-derivative, DMAC, in human colon cancer cells. Chem. Biol. Interact..

[17] Yadagiri B., Holagunda U.D., Bantu R., Nagarapu L., Kumar C.G., Pombala S., Sridhar B. (2014). Synthesis of novel building blocks of benzosuberone bearing coumarin moieties and their evaluation as potential anticancer agents. Eur. J. Med. Chem..

[18] Nasr T., Bondock S., Youns M. (2014). Anticancer activity of new coumarin substituted hydrazide-hydrazone derivatives. Eur. J. Med. Chem..

[19] Zou Q., Fang Y., Zhao Y., Zhao H., Wang Y., Gu Y., Wu F. (2013). Synthesis and *in vitro* photocytotoxicity of coumarin derivatives for one- and two-photon excited photodynamic therapy. J. Med. Chem..

[20] Barraja P., Spano V., Patrizia D., Carbone A., Cirrincione G., Vedaldi D., Salvador A., Viola G., Dall’acqua F. (2009). Pyrano[2,3-e]isoindol-2-ones, new angelicin heteroanalogues. Bioorg. Med. Chem. Lett..

[21] Lee J.H., Kim Y.G., Cho H.S., Ryu S.Y., Cho M.H., Lee J. (2014). Coumarins reduce biofilm formation and the virulence of escherichia coli o157:H7. Phytomedicine.

[22] Rea A., Tempone A.G., Pinto E.G., Mesquita J.T., Rodrigues E., Silva L.G., Sartorelli P., Lago J.H. (2013). Soulamarin isolated from *Calophyllum brasiliense* (Clusiaceae) induces plasma membrane permeabilization of *Trypanosoma cruzi* and mytochondrial dysfunction. PLoS Negl. Trop. Dis..

[23] Rehman S., Ikram M., Baker R.J., Zubair M., Azad E., Min S., Riaz K., Mok K., Rehman S.U. (2013). Synthesis, characterization, *in vitro* antimicrobial, and U2OS tumoricidal activities of different coumarin derivatives. Chem. Cent. J..

[24] Salas-Sarduy E., Cabrera-Munoz A., Cauerhff A., Gonzalez-Gonzalez Y., Trejo S.A., Chidichimo A., Chavez-Planes Mde L., Cazzulo J.J. (2013). Antiparasitic effect of a fraction enriched in tight-binding protease inhibitors isolated from the caribbean coral plexaura homomalla. Exp. Parasitol..

[25] Vila-Nova N.S., de Morais S.M., Falcao M.J., Alcantara T.T., Ferreira P.A., Cavalcanti E.S., Vieira I.G., Campello C.C., Wilson M. (2013). Different susceptibilities of *Leishmania* spp. Promastigotes to the *Annona muricata* acetogenins annonacinone and corossolone, and the *Platymiscium floribundum* coumarin scoparone. Exp. Parasitol..

[26] Sashidhara K.V., Kumar A., Dodda R.P., Krishna N.N., Agarwal P., Srivastava K., Puri S.K. (2012). Coumarin-trioxane hybrids: Synthesis and evaluation as a new class of antimalarial scaffolds. Bioorg. Med. Chem. Lett..

[27] Hemshekhar M., Sunitha K., Thushara R.M., Sebastin Santhosh M., Shanmuga Sundaram M., Kemparaju K., Girish K.S. (2013). Antiarthritic and antiinflammatory propensity of 4-methylesculetin, a coumarin derivative. Biochimie.

[28] Stefani H.A., Gueogjan K., Manarin F., Farsky S.H., Zukerman-Schpector J., Caracelli I., Pizano Rodrigues S.R., Muscara M.N., Teixeira S.A., Santin J.R. (2012). Synthesis, biological evaluation and molecular docking studies of 3-(triazolyl)-coumarin derivatives: Effect on inducible nitric oxide synthase. Eur. J. Med. Chem..

[29] Jang H.L., El-Gamal M.I., Choi H.E., Choi H.Y., Lee K.T., Oh C.H. (2014). Synthesis of tricyclic fused coumarin sulfonates and their inhibitory effects on LPS-induced nitric oxide and PGE2 productions in raw 264.7 macrophages. Bioorg. Med. Chem. Lett..

[30] Okuyama S., Minami S., Shimada N., Makihata N., Nakajima M., Furukawa Y. (2013). Anti-inflammatory and neuroprotective effects of auraptene, a citrus coumarin, following cerebral global ischemia in mice. Eur. J. Pharmacol..

[31] Sekiguchi H., Takabayashi F., Irie K., Murakami A. (2012). Auraptene attenuates gastritis via reduction of helicobacter pylori colonization and pro-inflammatory mediator production in c57bl/6 mice. J. Med. Food.

[32] Sekiguchi H., Irie K., Murakami A. (2010). Suppression of CD74 expression and helicobacter pylori adhesion by auraptene targeting serum starvation-activated Erk1/2 in NCI-n87 gastric carcinoma cells. Biosci. Biotechnol. Biochem..

[33] Bighetti A.E., Antonio M.A., Kohn L.K., Rehder V.L., Foglio M.A., Possenti A., Vilela L., Carvalho J.E. (2005). Antiulcerogenic activity of a crude hydroalcoholic extract and coumarin isolated from *Mikania laevigata* Schultz BIP. Phytomedicine.

[34] Goel R.K., Maiti R.N., Manickam M., Ray A.B. (1997). Antiulcer activity of naturally occurring pyrano-coumarin and isocoumarins and their effect on prostanoid synthesis using human colonic mucosa. Indian J. Exp. Biol..

[35] Choi W.S., Jang D.Y., Nam S.W., Park B.S., Lee H.S., Lee S.E. (2012). Antiulcerogenic activity of scoparone on Hcl/ethanol-induced gastritis in rats. J. Korean Soc. Appl. Biol..

[36] Choi W.S., Kim Y.S., Yang J.A., Lee Y.H., Park B.S., Lee S.E. (2011). Curative effects of extracts of hericium erinaceum hypha cultivated with *Artemisia capillaris* (HEAC) and their primary active compounds on rat liver disease. J. Korean Soc. Appl. Biol..

[37] Kitagawa H., Takeda F., Kohei H. (1986). A simple method for estimation of gastric mucus and effects of antiulcerogenic agents on the decrease in mucus during water-immersion stress in rats. Arzneim. Forsch..

[38] Kushima W., Hiruma-Lima C.A., Santos M.A., Viana E., Coelho-Ferreira M., Brito A.R.M.S. (2005). Gastroprotective activity of pradosia huberi on experimentally induced gastric lesions in rodents: Role of endogenous sulphydryls and nitric oxide. J. Ethnopharmacol..

[39] Zhang A., Sun H., Yuan Y., Sun W., Jiao G., Wang X. (2011). An *in vivo* analysis of the therapeutic and synergistic properties of chinese medicinal formula yin-chen-hao-tang based on its active constituents. Fitoterapia.

[40] Zhang A., Sun H., Wang X. (2014). Urinary metabolic profiling of rat models revealed protective function of scoparone against alcohol induced hepatotoxicity. Sci. Rep..

[41] Kang J.W., Kim D.W., Choi J.S., Kim Y.S., Lee S.M. (2013). Scoparone attenuates d-galactosamine/lipopolysaccharide-induced fulminant hepatic failure through inhibition of toll-like receptor 4 signaling in mice. Food Chem. Toxicol..

[42] Atmaca M., Bilgin H.M., Obay B.D., Diken H., Kelle M., Kale E. (2011). The hepatoprotective effect of coumarin and coumarin derivates on carbon tetrachloride-induced hepatic injury by antioxidative activities in rats. J. Physiol. Biochem..

[43] Niu N., Li B., Hu Y., Li X., Li J., Zhang H. (2014). Protective effects of scoparone against lipopolysaccharide-induced acute lung injury. Int. Immunopharmacol..

[44] Choi Y.H., Yan G.H. (2009). Anti-allergic effects of scoparone on mast cell-mediated allergy model. Phytomedicine.

[45] Kim J.K., Kim J.Y., Kim H.J., Park K.G., Harris R.A., Cho W.J., Lee J.T., Lee I.K. (2013). Scoparone exerts anti-tumor activity against DU145 prostate cancer cells via inhibition of stat3 activity. PLoS One.

[46] Kielbus M., Skalicka-Wozniak K., Grabarska A., Jeleniewicz W., Dmoszynska-Graniczka M., Marston A., Polberg K., Gawda P., Klatka J., Stepulak A. (2013). 7-Substituted coumarins inhibit proliferation and migration of laryngeal cancer cells *in vitro*. Anticancer Res..

[47] Mogana R., Adhikari A., Debnath S., Hazra S., Hazra B., Teng-Jin K., Wiart C. (2014). The antiacetylcholinesterase and antileishmanial activities of canarium patentinervium MIQ. BioMed Res. Int..

[48] Yang Y.J., Lee H.J., Huang H.S., Lee B.K., Choi H.S., Lim S.C., Lee C.K., Lee M.K. (2009). Effects of scoparone on dopamine biosynthesis and l-dopa-induced cytotoxicity in PC12 cells. J. Neurosci. Res..

[49] Lee S.H., Jang H.D. (2015). Scoparone attenuates RANKL-induced osteoclastic differentiation through controlling reactive oxygen species production and scavenging. Exp. Cell Res..

[50] Anand P., Singh B., Singh N. (2012). A review on coumarins as acetylcholinesterase inhibitors for Alzheimer’s disease. Bioorg. Med. Chem..

[51] Allen A., Flemstrom G. (2005). Gastroduodenal mucus bicarbonate barrier: Protection against acid and pepsin. Am. J. Physiol..

[52] Yang H., Xu L.N., Sui Y.J., Liu X., He C.Y., Fang R.Y., Liu J., Hao F., Ma T.H. (2011). Stimulation of airway and intestinal mucosal secretion by natural coumarin CFTR activators. Front. Pharmacol..

[53] Greaves P. (2007). Histopathology of Preclinical Toxicity Studies: Interpretation and Relevance in Drug Safety Evaluation.

[54] Schmidt L.E., Dalhoff K. (2002). Food-drug interactions. Drugs.

[55] Bushra R., Aslam N., Khan A.Y. (2011). Food-drug interactions. Oman Med. J..

[56] Obach R.S., Zhang Q.Y., Dunbar D., Kaminsky L.S. (2001). Metabolic characterization of the major human small intestinal cytochrome p450s. Drug Metab. Dispos..

[57] Kanazu T., Yamaguchi Y., Okamura N., Baba T., Koike M. (2004). Model for the drug-drug interaction responsible for Cyp3a enzyme inhibition. II: Establishment and evaluation of dexamethasone-pretreated female rats. Xenobiot. Fate Foreign Compd. Biol. Syst..

[58] Rettie A.E., Jones J.P. (2005). Clinical and toxicological relevance of Cyp2c9: Drug-drug interactions and pharmacogenetics. Annu. Rev. Pharmacol. Toxicol..

[59] Kirchheiner J., Brockmoller J. (2005). Clinical consequences of cytochrome p450 2c9 polymorphisms. Clin. Pharmacol. Ther..

[60] Gurley B.J., Gardner S.F., Hubbard M.A., Williams D.K., Gentry W.B., Cui Y., Ang C.Y. (2002). Cytochrome p450 phenotypic ratios for predicting herb-drug interactions in humans. Clin. Pharmacol. Ther..

[61] Zhou S., Lim L.Y., Chowbay B. (2004). Herbal modulation of p-glycoprotein. Drug Metab. Rev..

[62] Wittkowsky A.K. (2001). Drug interactions update: Drugs, herbs, and oral anticoagulation. J. Thromb. Thromb..

[63] Kayser O., Kolodziej H. (1997). Antibacterial activity of extracts and constituents of pelargonium sidoides and pelargonium reniforme. Planta Med..

[64] Satyanarayana P., Kumar K.A., Singh S.K., Rao G.N. (2001). A new phorbol diester from aleurites moluccana. Fitoterapia.

[65] Lee S., Kim K.S., Shim S.H., Park Y.M., Kim B.K. (2003). Constituents from the non-polar fraction of *Artemisia apiacea*. Arch. Pharm. Res..

[66] Uddin S.J., Shilpi J.A., Middleton M., Byres M., Shoeb M., Nahar L., Sarker S.D. (2007). Swarnalin and *cis*-swarnalin, two new tetrahydrofuran derivatives with free radical scavenging activity, from the aerial parts of *Cuscuta reflexa*. Nat. Prod. Res..

[67] Saeed M.A., Sabir A.W. (2008). Irritant and cytotoxic coumarins from *Angelica glauca* edgew roots. J. Asian Nat. Prod. Res..

[68] Lee S.J., Kim H.M., Lee J.M., Park H.S., Lee S. (2008). Artemisterol, a new steryl ester from the whole plant of *Artemisia apiacea*. J. Asian Nat. Prod. Res..

[69] Traore M., Jaroszewski J.W., Olsen C.E., Ouedraogo J.B., Pierre G.I., Nacoulma O.G., Guiguemde T.R., Christensen S.B. (2008). A new oxygenated ursane derivative from *Canthium multiflorum*. Planta Med..

[70] Li Q.J., Wang M.L., Yang X.S., Ma L., Hao X.J. (2013). Two new coumarin glycosides from *Chimonanthus nitens*. J. Asian Nat. Prod. Res..

[71] Kolodziej H. (2007). Fascinating metabolic pools of pelargonium sidoides and pelargonium reniforme, traditional and phytomedicinal sources of the herbal medicine umckaloabo. Phytomedicine.

[72] Estevez-Braun A., Estevez-Reyes R., Moujir L.M., Ravelo A.G., Gonzalez A.G. (1994). Antibiotic activity and absolute configuation of 8s-heptadeca-2(z),9(z)-diene-4,6-diyne-1,8-diol from bupleurum salicifolium. J. Nat. Prod..

[73] Mbwambo Z.H., Lee S.K., Mshiu E.N., Pezzuto J.M., Kinghorn A.D. (1996). Constituents from the stem wood of euphorbia quinquecostata with phorbol dibutyrate receptor-binding inhibitory activity. J. Nat. Prod..

[74] Rollinger J.M., Schuster D., Danzl B., Schwaiger S., Markt P., Schmidtke M., Gertsch J., Raduner S., Wolber G., Langer T. (2009). *In silico* target fishing for rationalized ligand discovery exemplified on constituents of *Ruta graveolens*. Planta Med..

[75] Gao W., Li Q., Chen J., Wang Z., Hua C. (2013). Total synthesis of six 3,4-unsubstituted coumarins. Molecules.

