# Supplementary material for: Gastroprotective Efficacy and Safety Evaluation of Scoparone Derivatives on Experimentally Induced Gastric Lesions in Rodents
Source: Nutrients. 2015 Mar 13;7(3):1945–64. doi: 10.3390/nu7031945 (PMC4377892; doi:10.3390/nu7031945)
Supplement: Supplementary File 1 [file nutrients-07-01945-s001.docx]

**Supplementary Information**


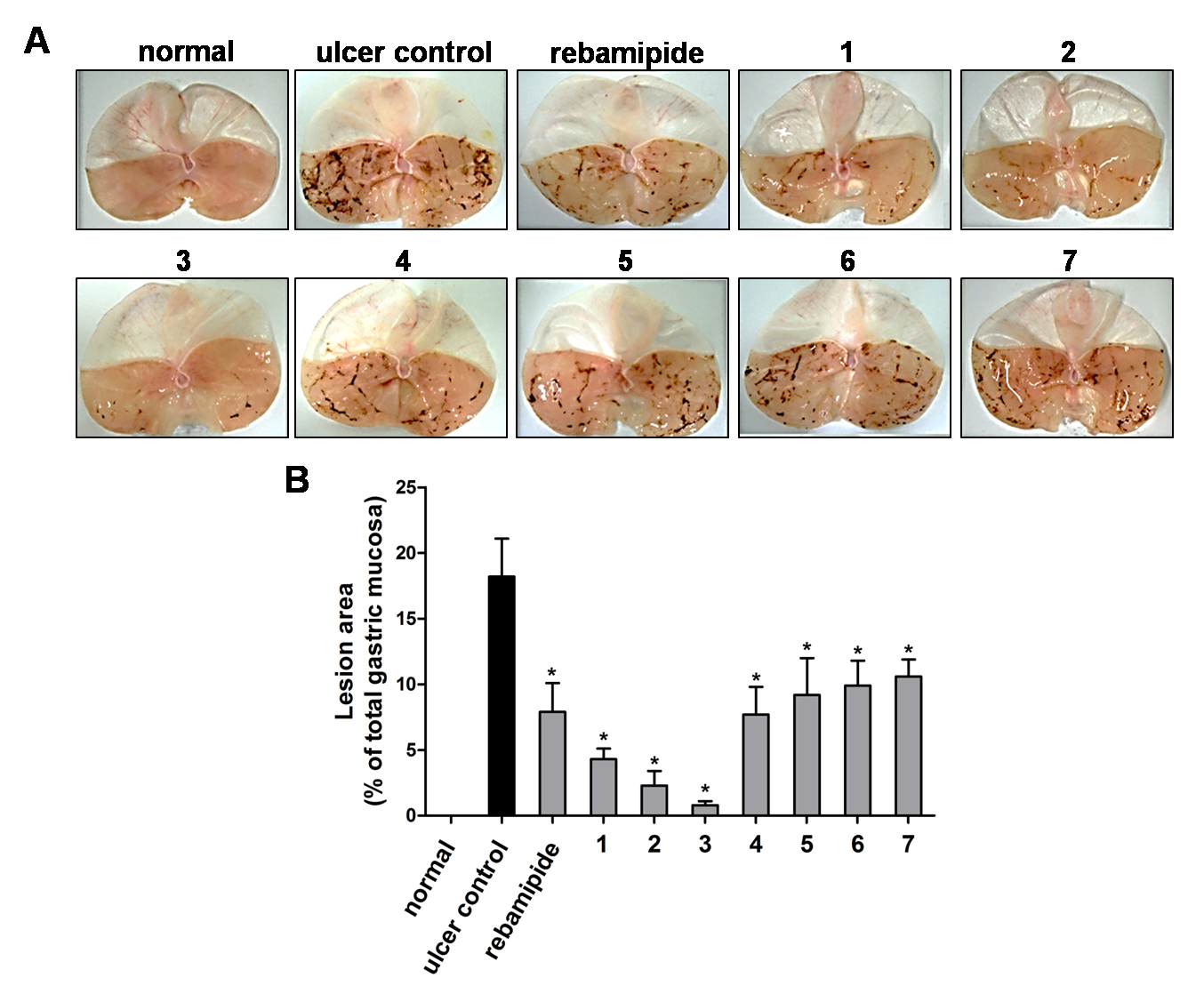


**Figure S1.** Effect of scoparone and its synthetic derivatives on indomethacin-induced gastric ulcers. (**A**) Rats treated with the normal saline (vehicle) alone show no lesion on the gastric mucosal surface (normal). Rats pretreated with normal saline followed by oral administration with indomethacin. Indomethacin produced visible hemorrhage necrosis of gastric mucosa (ulcer control). Rats pretreated with a 40 mg/kg dose of rebamipide, scoparone (**1**), 5,6,7-trimethoxycomarin (**2**), 6,7,8-trimethoxycoumarin (**3**), 6-methoxy-7,8-methylenedioxycoumarin (**4**), 6-methoxy-7,8-(1-methoxy)-methylenedioxycoumarin (**5**), 6,7-methylenedioxycoumarin (**6**), or 6,7-(1-methoxy)-methylenedioxycoumarin (**7**), respectively, followed by oral administration of indomethacin. Stomachs were dissected and imaged by bright field microscopy. Representative images of each experimental group are shown; (**B**) Gastric ulcer lesion area was quantified. Data shown as mean ± SEM of
(*n* = 8). ***** *p* < 0.05 *vs.* ulcer control as determined by Student’s *t*-test.

**Table S1.** Evaluation of scoparone derivatives on organ weight and mortality in mice.

| **Treatment** | **Relative Organ Weight (% body weight)** | | | | | **Mortality** |
| --- | --- | --- | --- | --- | --- | --- |
|  | **Liver** | **Kidney** | **Spleen** | **Heart** | **Lung** |  |
| *Male* |  |  |  |  |  |  |
| Vehicle control | 5.48 ± 0.22 | 1.59 ± 0.02 | 0.38 ± 0.04 | 0.52 ± 0.02 | 0.70 ± 0.05 | 0/10 |
| 5,6,7-trimethoxycoumarin (**2**) | 5.84 ± 0.23 | 1.56 ± 0.05 | 0.36 ± 0.08 | 0.46 ± 0.07 | 0.68 ± 0.05 | 0/10 |
| 6,7,8-trimethoxycoumarin (**3**) | 4.92 ± 0.24 | 1.58 ± 0.06 | 0.40 ± 0.07 | 0.50 ± 0.02 | 0.69 ± 0.06 | 0/10 |
| *Female* |  |  |  |  |  |  |
| Vehicle control | 6.60 ± 0.58 | 1.35 ± 0.06 | 0.44 ± 0.05 | 0.55 ± 0.04 | 0.77 ± 0.06 | 0/10 |
| 5,6,7-trimethoxycoumarin (**2**) | 4.21 ± 0.64 | 1.22 ± 0.09 | 0.45 ± 0.09 | 0.47 ± 0.05 | 0.69 ± 0.07 | 0/10 |
| 6,7,8-trimethoxycoumarin (**3**) | 6.86 ± 0.78 | 1.34 ± 0.05 | 0.48 ± 0.10 | 0.54 ± 0.05 | 0.79 ± 0.06 | 0/10 |

Values are expressed as mean ± SEM (*n* = 10). There are no significant differences between groups as determined by Student’s *t*-test.

© 2015 by the authors; licensee MDPI, Basel, Switzerland. This article is an open access article distributed under the terms and conditions of the Creative Commons Attribution license (http://creativecommons.org/licenses/by/4.0/).
